# Motherwort Injection for Preventing Uterine Hemorrhage in Women With Induced Abortion: A Systematic Review and Meta-Analysis of Randomized Evidence

**DOI:** 10.3389/fphar.2022.916665

**Published:** 2022-07-21

**Authors:** Xue Xinyu, Tang Xintong, Li Youping, Wan Feng, Yu Jiajie

**Affiliations:** ^1^ Chinese Evidence-based Medicine Center, West China Hospital, Sichuan University, Chengdu, China; ^2^ State Key Laboratory of Southwestern Chinese Medicine Resources, Chengdu University of Traditional Chinese Medicine, Chengdu, China,

**Keywords:** induced abortion, motherwort injection, Oxytocin, uterine hemorrhage, meta-analysis, systematic review, randomized controlled trials

## Abstract

**Objective:** Motherwort injection (MI) is a modern patented injection extracted from motherwort (Leonurus japonicus Hoult). Empirical studies and systematic reviews have shown the benefits of motherwort injection for preventing postpartum hemorrhage after vaginal delivery and cesarean section. This study was conducted to explore the efficacy and safety of motherwort injection for women with the prevention of post-abortion uterine hemorrhage.

**Methods:** A comprehensive literature search was conducted to identify RCTs regarding the effect of the use of motherwort injection in women after abortion. Data from trials were pooled by meta-analysis and a random-effects model was used to calculate the summarized relative risks (RRs) and their 95% confidence intervals (CIs). The grading of recommendations assessment, development, and evaluation (GRADE) methodology was used to access the quality of the evidence.

**Results:** Nine trials with a total of 1,675 participants were identified. Overall, motherwort injection combined with oxytocin compared to oxytocin had a significantly lower blood loss within 2 hours (MD = −50.00, 95% CI −62.92 to −37.08, very low quality); lower blood loss within 24 h (MD = −50.00, 95% CI −62.92 to −37.08, very low quality); however, there was no significant difference between motherwort injection and oxytocin (24 h: MD: 0.72, 95% CI −7.76 to 9.20; 48 h: MD: −0.01, 95% CI −11.35 to 11.33; 72 h: MD: −1.12, 95% CI −14.39 to 12.15, very low quality). Compared with oxytocin or no intervention, both motherwort injection and motherwort injection combined with oxytocin had a significantly decreased duration of blood loss (MI vs. O: MD −2.59, 95% CI −4.59 to −0.60, very low quality; MI + O vs. O: MD −2.62, 95% CI -3.02 to −2.22, very low quality; MI + O vs. No intervention: MD: −1.80, 95% CI −2.28 to −1.33, low quality). Seven of nine included trials reported adverse event outcomes. Three cases were found in the motherwort injection group, and five induced abortion syndromes were found in the motherwort injection plus oxytocin group. 29 adverse events were reported in the oxytocin group instead. The recovery time of normal menstruation after abortion was significantly earlier in the group using motherwort injection compared with oxytocin (MDs −3.77, 95% CI −6.29 to −1.25, very low quality), and the endometrial thickness in the motherwort injection group was significantly different from that in the oxytocin group (MD: 2.24, 95% CI 1.58 to 2.90, very low quality).

**Conclusion:** The results of this meta-analysis indicate prophylactic use of motherwort injection may reduce the risk of uterine hemorrhage in women after abortion, and more high-quality research is needed to confirm the efficacy and safety of motherwort injection in preventing uterine hemorrhage after abortion.

**Systematic Review Registration:** https://www.crd.york.ac.uk/PROSPERO/display_record.php?RecordID=274153, identifier CRD42021274153

## 1 Introduction

An estimated 205 million pregnancies occur each year worldwide, with 20% being terminated by induced abortion. Medication and surgery are both highly effective methods for induced abortion and are determined by the gestational age of the embryo or tutus. Abortion is one of the safest procedures when properly done, but unsafe abortion is associated with significant morbidity (Organization., 2021). Hemorrhage is the common consequence of misled management of abortion care and risks including retained tissues, uterine injury, uterine atony, and vaginal laceration are related to uterine bleeding after an abortion (Kerns and Steinauer, 2013; Perriera et al., 2017). The administration of uterotonic agents, such as oxytocin, misoprostol, and methylergonovine, is recommended to prevent hemorrhage after induced abortion if the uterus is atonic (Evensen et al., 2017; Bienstock et al., 2021; Steinauer and Patil, 2021). However, excessive use of oxytocin and misoprostol may cause high fever, shaking, chills, vomiting, hypertension, and other complications of toxicity (Widmer et al., 2010; Cleland et al., 2013).

Motherwort injection (MI) is a modern patented injection made from aqueous extracts of motherwort (Leonurus japonicus Hoult), which is a traditional Chinese herb used by thousands for gynecological conditions in China (Lulin et al., 2019). Pharmacological studies have shown that the active ingredients of motherwort injection (i.e. alkaloid, leonurine) could significantly facilitate hemostatic outcomes by promoting uterine contraction and blocking the uterine spiral vessels (Jian et al., 2005; Ojewole, 2005; Xiaoju, 2009; Huizhen et al., 2021). Moreover, motherwort injection works on the lower uterus without the receptor saturation effect, which reduces the risk of adverse events caused by the excessive use of uterotonic agents (Wenjing, 2022). Therefore, the prophylactic use of motherwort injection with or without oxytocin has been widely applied in Chinese tertiary hospitals to prevent postpartum and postabortion hemorrhage since 2005.

Empirical studies (Jianhua et al., 2009; Wei et al., 2016) and systematic reviews (Wenwen et al., 2018; Jiajie et al., 2019) have illustrated the benefits of motherwort injection for preventing PPH (postpartum hemorrhage) after vaginal delivery and cesarean section. Meanwhile, some trials have been published to explore the effect of motherwort injection on women after induced abortion and the findings were inconsistent. Therefore, we conducted a systematic review of randomized trials to determine the efficacy and safety of motherwort injection compared to oxytocin in women with induced abortion.

## 2 Materials and Method

This systematic review has been registered in the PROSPERO database (CRD42021274153) and reported according to The Preferred Reporting Items for Systematic Reviews and Meta-Analysis (PRISMA) statement guidelines (Page et al., 2021) (Supplementary Material S1).

### 2.1 Eligibility Criteria

Randomized control trials were eligible if they met the following criteria: 1) Participants: pregnant women anticipating an induced abortion; 2) Intervention: motherwort injection given by any route of administration and dose used alone or in combination with oxytocin; 3) Control: no intervention or oxytocin alone; 4) Outcomes measures: duration of uterine hemorrhage, the volume of blood loss, adverse events, the recovery time of normal menstruation, and endometrial thickness. We excluded trials reporting blood loss as a categorical variable due to a lack of classification criteria.

### 2.2 Data Source and Search Strategy

Relevant studies were identified from PubMed, EMbase, Cochrane Central Register of Controlled Trials (CENTRAL), Chinese National Knowledge Infrastructure Database (CNKI) and WanFang database inception to December 2021, updated to May 2022. An information expert was consulted to optimize our search strategies (Supplementary Material S2). ClinicalTrial.gov and Chinese Clinical Trial Registry were searched to identify unpublished studies, and the reference lists of included trials were searched for additional eligible studies. The search strategy was based on Mesh terms and their variants. No restriction in language was applied.

We also contacted a content expert and industry representatives and searched conference abstracts for additional information.

### 2.3 Data Selection and Data Extraction

Two reviewers (Xue XY and Tang XT) used predefined, pilot-tested forms to screen studies for eligibility, independently screened titles/abstracts, and full text of potentially eligible articles. They independently assessed the risk of bias, quality of evidence and extracted data. If necessary, discrepancies were resolved through discussion. We collected information regarding study characteristics (sample size, publication year, author name, affiliation, and multicenter study), participants’ characteristics (age, gestational week, and risk factors), interventions (dosage, timing, injection site, and duration of treatment), and outcomes (duration of hemorrhage, blood loss, adverse events, recovery time of normal menstruation, and endometrial thickness).

### 2.4 Risk of Bias Assessment

We assessed the risk of bias using the revised Cochrane Risk of Bias tool (Higgins et al., 2011; Akl et al., 2012) in our published study. The items included randomization sequence generation, allocation concealment, blinding of patients and personnel, or outcome assessors, infrequent missing outcome data, selective outcome reporting, and funding resources. The risk of bias for each item will be classified into low risk, high risk, and unclear. The risk of bias for each item will be classified into low risk, high risk, and unclear. The options for an overall risk of bias are the same as for individual items and are based on the following criteria: 1) trials were judged to be at low risk of bias if all items were assessed as low risk; 2) to be at high risk in at least one item assessed as high risk, or multiple items were assessed as an unclear risk; 3) to be an unclear risk in at least one item assessed as unclear but not to be at high risk for any item (Higgins et al., 2022).

### 2.5 Data Analysis and Rating Quality of Evidence

The data were pooled using a random-effects model for potential heterogeneity among studies when two or more studies assessed the same outcome. Heterogeneity among studies was assessed by Cochran’s Q test and the I^2^ statistic. We expressed dichotomous data as risk ratio (RR) with 95% confidence intervals (CIs) and continuous data as mean differences (MDs) with 95% CIs. If the trial was comparing three groups, we separately analyzed the data in terms of their interventions. The intervention arm was included twice in the analysis; however, this was related to only one trial, and this double inclusion would not influence the outcomes. Subgroup analyses were performed based on the type of administration (immediate administration versus consecutive administration) and risk for hemorrhage after abortion (high risk vs. moderate risk vs. low risk) when applicable. We summarized the adverse event data from all included studies and qualitatively described the data for rare data. Publication bias was assessed using Egger test plots when ten or more studies were available (Higgins JPT et al., 2022). RevMan 5.4 software was used for meta-analysis. We used the grading of recommendations assessment, development, and evaluation (GRADE) methodology to assess the quality of the evidence (Guyatt et al., 2008).

## 3 Results

### 3.1 Search Results

**FIGURE 1:**
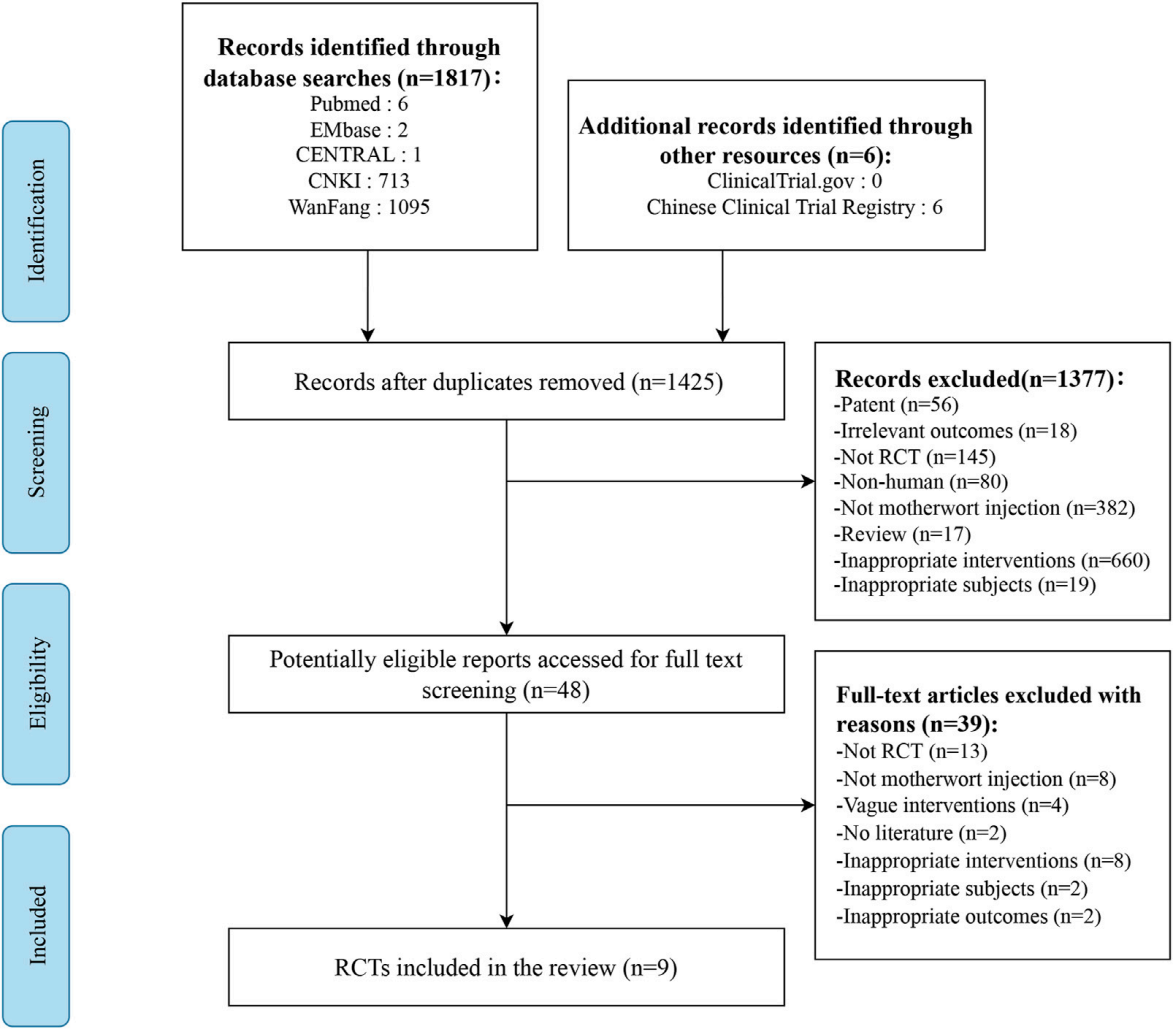
Screening flow chart.

Seven databases were screened yielding a total of 1823 studies. After removing duplicates and title and abstract screening, 48 studies were selected for a full-text review. Of 48 potentially relevant studies, 37 were excluded (e.g., studies were not properly randomized, or did not report relevant outcomes, etc.,) and two were abstracts without outcome measures. Finally, nine studies involving 1,675 women were included in the systematic review. The selection process is listed in Figure 1.

### 3.2 Study Characteristics

One multicenter RCT was identified and the remaining RCTs were single centers. All studies were reported in Chinese except one (Wanting et al., 2020) in English. These studies were all conducted in China between 2006 and 2019, from 60 to 366, and the characteristics of included studies are shown in Table 1. The mean age of pregnant women was 26.43 (SD:6.72), the mean pregnancy time was 2.31 (SD:1.10), and the mean gestation was 54.18 (SD:10.62) days. Two studies (Juan, 2016; Yun and Dengyu, 2016) evaluated motherwort injection combined with oxytocin versus oxytocin alone, four studies (Jiaogui, 2010; Li and Lixia, 2012; Yingjie and Junling, 2016; Yunyan, 2019) compared motherwort injection with oxytocin, and two studies (Yimei et al., 2008; Wanting et al., 2020) compared motherwort injection to no intervention. There was one trial (Yanxia, 2012) with multiple arms (MI vs. O vs. None).

**TABLE 1:** Characteristics of included studies.

Study	Intervention T/C	Participants T/C	Age (year) Rang/mean	Pregnancy time Rang/mean	Gestation (DAY) Rang/Mean	Injection site T/C	Usage T/C	Dosage T/C
Peng 2016	M + O	115	27.60 (5.30)	—	62.30 (5.80)	Intramuscular	Immediate	2 ml(M)+ 1 ml(O)
	O	115	27.60 (5.30)	—	62.30 (5.80)	Intramuscular	Immediate	1 ml(O)
He 2016	M + O	90	26.03 (6.21)	2.12 (0.35)	57.33 (9.94)	Intramuscular(M)	Immediate	2 ml(M)+Unclear(O)
						Cervical(O)		
	O	90	25.36 (6.78)	2.03 (0.32)	56.84 (11.76)	Cervical	Immediate	Unclear
Yuan 2010	M	90	23.95 (9.91)	—	53.54 (9.11)	Intramuscular	Continuous	3 ml(M)
	O	90	24.14 (9.98)	—	53.69 (8.86)	Intramuscular	Continuous	3 ml(O)
Huang 2012b	M	129	17–39	—	—	Intramuscular	Immediate	2 ml(M)
	O	131	17–39	—	—	Intramuscular	Immediate	2 ml(O)
Ouyang 2019	M	30	28.24 (3.63)	2.08 (0.33)	42.13 (1.56)	Intramuscular	Immediate	2 ml(M)
	O	30	29.51 (3.45)	2.14 (0.43)	41.87 (1.35)	Intramuscular	Immediate	1 ml(O)
Zhang 2016	M	30	29.10 (7.10)	3.19 (0.27)	41.10 (1.70)	Intramuscular	Immediate	2 ml(M)
	O	30	27.20 (6.70)	3.06 (0.14)	42.40 (1.80)	Intramuscular	Immediate	1 ml(O)
Yuan 2012	M	43	26.58 (6.09)	2.81 (1.29)	43.88 (6.53)	Cervical + Intramuscular	Continuous	2 ml(M)
	O	44	27.59 (6.62)	3.00 (1.53)	45.86 (6.78)	Cervical + Intramuscular	Continuous	2 ml(O)
Xia 2020	M	188	26.86 (5.89)	2.24 (1.33)	—	Intramuscular	Continuous	8 ml(M)
	No Intervention	178	25.89 (5.50)	2.12 (1.26)	—	None	None	None
Huang 2012a	M	129	17–39	—	—	Intramuscular	Immediate	2 ml(M)
	No Intervention	102	17–39	—	—	None	None	None
Zhang 2008	M	100	—	—	—	Intramuscular	Immediate	1 ml(M)/2 ml(M)
	No Intervention	50	—	—	—	None	None	None

M, motherwort injection; O, oxytocin.

### 3.3 Risk of Bias Within Studies

Among these nine trials, two (Yingjie and Junling, 2016; Wanting et al., 2020) adequately generated random sequences by random number table or computer; none of them clearly stated how to conceal the random sequence and blind the participants, health care providers, or outcome assessors; none of them reported selective outcomes, and one trial reported the funding resource. Table 2 contains detailed results of the assessment. Overall, each of the included studies assessed the risk of bias to be unclear.

**TABLE 2:** Risk of bias.

Study	Randomization	Concealed allocation	Blinding		Integrity of result	Selective reporting	Other bias (funding resources)
			For participants	For outcome assessment			
Peng 2016	Only mentioned	NR	NR	NR	Complete	NR	NR
Yuan 2010	Only mentioned	NR	NR	NR	Complete	NR	NR
Huang 2012	Only mentioned	NR	NR	NR	Complete	NR	NR
Ouyang 2019	Only mentioned	NR	NR	NR	Complete	NR	NR
Zhang 2016	Random number table	NR	NR	NR	Complete	NR	NR
He 2016	Only mentioned	NR	NR	NR	Complete	NR	NR
Yuan 2012	Only mentioned	NR	NR	NR	7 cases in MI group had incomplete records of bleeding, and 6 cases in the oxytocin group had incomplete records	NR	NR
Xia 2020	Random number table	NR	NR	NR	Completed in 398 patients (201 patients assigned to MI and 199 assigned to no-treatment), and 366 patients completed the follow-up assessment (188 patients assigned to LHI and 178 assigned to no-treatment)	NR	Supported by the science and technology support project of Sichuan province and the science and technology achievements transformation demonstration project of Sichuan province
Zhang 2008	Only mentioned	NR	NR	NR	Complete	NR	NR

### 3.4 Outcome measures

#### 3.4.1 Blood Loss

##### 3.4.1.1Motherwort Injection vs. Oxytocin

Only one RCT (Li and Lixia, 2012) involving 87 participants reported blood loss within 24 h, 48 h, and 72 h. The data from this trial showed no significant difference between motherwort injection and oxytocin in all three assessments (24 h: MD: 0.72, 95% CI −7.76 to 9.20; 48 h: MD: −0.01, 95% CI −11.35 to 11.33; 72 h: MD: −1.12, 95% CI −14.39 to 12.15, very low quality).

##### 3.4.1.2 Motherwort Injection Plus Oxytocin vs. Oxytocin

One trial (*n* = 230) (Juan, 2016) compared motherwort injection to oxytocin reporting blood loss within 2 hours and 24 h. There was a significant decrease in blood loss in the combined group compared to oxytocin alone (2 h: MD: −50.00, 95% CI −62.92 to −37.08; 24 h: MD: −50.00, 95% CI −62.92 to -37.08, very low quality).

### 3.5 Duration of Blood Loss (days)

#### 3.4.1 Motherwort Injection vs. Oxytocin

**FIGURE 2:**
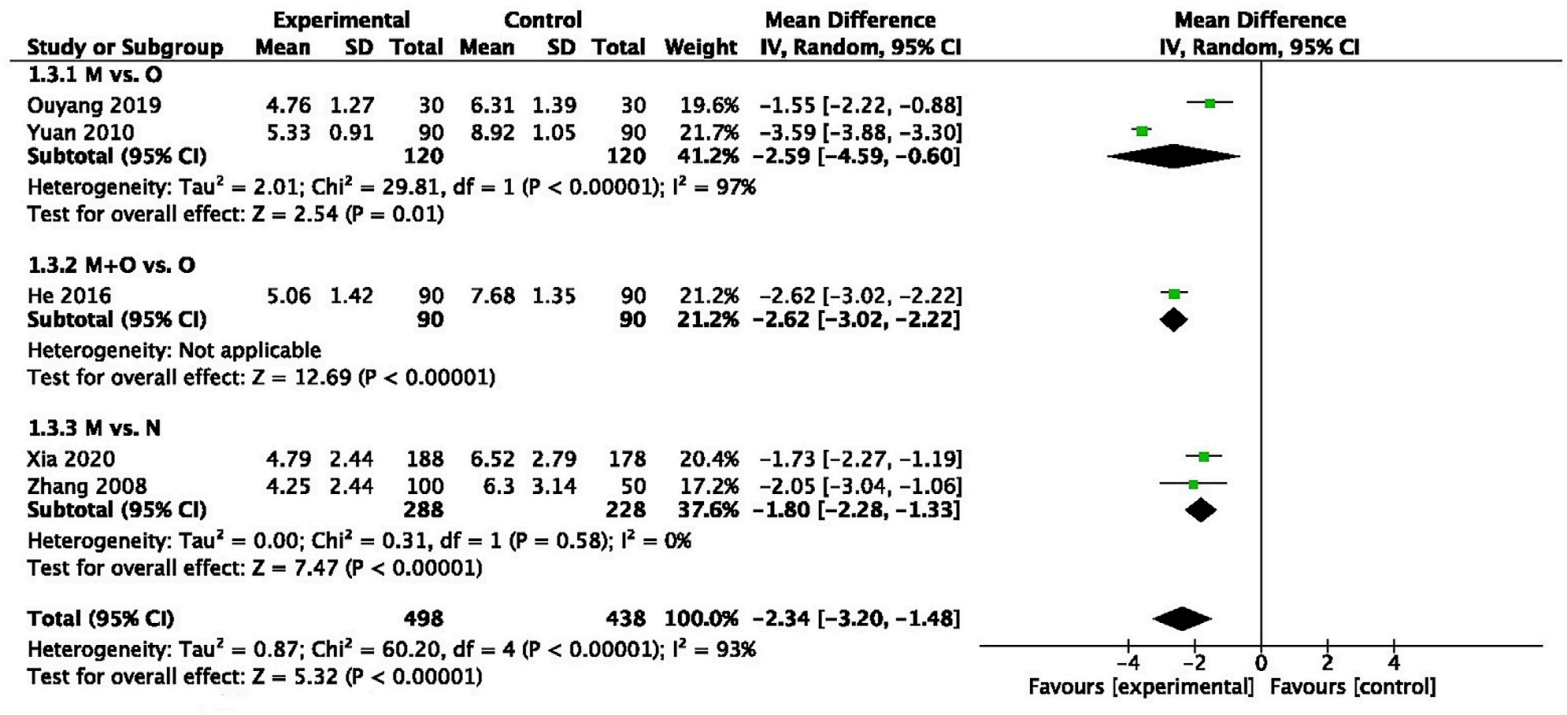
Duration of blood loss.

There was statistically significant heterogeneity among two trials (*n* = 240, I^2^ = 97%) (Jiaogui, 2010; Yunyan, 2019), and pooled data demonstrated that motherwort injection significantly decreased the duration of blood loss compared to oxytocin (MD −2.59, 95% CI −4.59 to −0.60, very low quality) (Figure 2).

#### 3.5.2 Motherwort Injection Plus Oxytocin vs. Oxytocin

We also observed a significantly decreased duration of blood loss in the combination administration group in one trial (MD -2.62, 95% CI −3.02 to −2.22, very low quality) (Yun and Dengyu, 2016) (Figure 2).

#### 3.5.3 Motherwort Injection vs. no Intervention

Two trials (*n* = 516) (Yimei et al., 2008; Wanting et al., 2020) collected data for the duration of blood loss, and there was a significant reduction in days after motherwort injection (MD: −1.80, 95% CI −2.28 to −1.33, low quality) (Figure 2).

### 3.6 Adverse Events

Seven of nine included trials commented on adverse event outcomes, and three of them (Jiaogui, 2010; Yanxia, 2012; Juan, 2016) stated none had occurred. The remaining four trials (Yingjie and Junling, 2016; Yun and Dengyu, 2016; Yunyan, 2019; Wanting et al., 2020) reported adverse events, three cases (mild erythema, nausea and vomiting) (1.2%, 3/248) were found in the motherwort injection group, and five induced abortion syndromes were found in the motherwort injection plus oxytocin group (5.5%, 5/90). 29 adverse events (e.g., induced abortion syndromes, nausea, vomiting, infection, etc.) were reported in the oxytocin group instead (19.3%, 29/150) (Table 3).

**TABLE 3:** Adverse events.

Study	Intervention	Participants	Adverse events
	T/C	T/C	T/C
Peng 2016	M + O	115	None
	O	115	None
He 2016	M + O	90	5 induced abortion syndromes
	O	90	19 induced abortion syndromes
Yuan 2010	M	90	None
	O	90	None
Ouyang 2019	M	30	None
	O	30	1 gastrointestinal discomfort
Zhang 2016	M	30	1 nausea, 1 vomiting
	O	30	1 allergy, 1 nausea, 3 vomiting, 1 diarrhea, 3 infections
Xia 2020	M	188	1 mild erythema
	No Intervention	178	None
Huang2012	M	129	None
	O	131	None
	No Intervention	102	None

### 3.7 Recovery Time of Normal Menstruation

#### 3.7.1 Motherwort Injection vs. Oxytocin (days)

Only one trial (*n* = 60) (Yunyan, 2019) comparing motherwort injection with oxytocin discussed the recovery time of normal menstruation. The data showed significantly earlier recovery of normal menstruation in the motherwort injection group than those in the oxytocin group (MDs −3.77, 95% CI −6.29 to −1.25, very low quality).

### 3.8 Endometrial Thickness

#### 3.8.1 Motherwort Injection vs. Oxytocin (mm)

Only one RCT (Yunyan, 2019) involving 60 participants reported endometrial thickness after abortion. The data from this trial showed a significant difference in endometrial thickness after abortion between the two groups (MD: 2.24, 95% CI 1.58 to 2.90, very low quality).

### 3.9 Subgroup Analysis and Publication Bias

We failed to conduct the subgroup analysis and assess publication bias for the small number of studies included in each outcome measure.

## 4 Discussion

This review brings all randomized evidence together to assess the effect of motherwort injection for postabortion hemorrhage. Compared with oxytocin, a significant benefit of prophylactic use of motherwort injection with or without oxytocin was reported for four outcomes: duration of blood loss, adverse events, and the recovery time of normal menstruation and endometrial thickness. In contrast, only two trials reported blood loss as a continuous variable and no significant difference was found between motherwort injection and oxytocin. Considering the low and very low methodological quality, small sample size and the observed difference between groups, this evidence supporting the use of prophylactic use of motherwort injection for women with postabortion hemorrhage must be generalized with caution.

Atony of the uterine body or fundus is a common cause of postabortion hemorrhage (Gill et al., 2022), and the risk of bleeding is associated with prior cesarean section, history of obstetrical hemorrhage, increasing maternal age, gestational age and obesity (Upadhyay et al., 2015; Kerns et al., 2019). Uterotonic agents are a priority protocol for the prevention and treatment of postabortion hemorrhage in women with uterine atony, including methylergonovine, misoprostol and oxytocin. However, little evidence exists to recommend starting with a particular agent. Motherwort injection, approved for marketing in 1971 by the Chinese FDA, has been used for stopping bleeding and regulating menstruation for decades (Yulin et al., 2018). Modern pharmacological studies have demonstrated that the active ingredients of motherwort injection, such as leonurine and stachydrine (Yulin et al., 2018), could exhibit angiogenic activity and have an excitatory effect on the uterus, without adverse effects such as elevated blood pressure (Dan et al., 2013; Xiaofei et al., 2014; Rebonato et al., 2016; Juan et al., 2018; Liefang, 2021; Qiang et al., 2021). These active ingredients can also dilate blood vessels and protect the cardiovascular system (Chengping et al., 2019), and will not affect women’s temperature and respiration (Yanfang et al., 2017). In addition, oxytocin is a polypeptide hormone with uterine contraction and has a rapid onset of action (Aiqun et al., 2007), while motherwort injection causes contractions for a longer period than oxytocin after injection into the uterine wall (half-life is 6 h) with a relatively slow onset of action. Therefore, the additional use of motherwort injection on oxytocin has been widely applied in routine clinical practice.

Our published SRs (Wenwen et al., 2018; Jiajie et al., 2019) have suggested the preferable outcomes of motherwort injection for preventing PPH (postpartum hemorrhage) after vaginal delivery and cesarean section. However, even with wide application in clinical settings, limited studies have been conducted to discuss the effect of motherwort injection on a postabortion hemorrhage. To the best of our knowledge, this is the first systematic review and meta-analysis to address the effect of motherwort injection on a postabortion hemorrhage.

### 4.1 Limitations

We conducted a comprehensive systematic review including all published RCTs with rigorous methods to evaluate the effect of motherwort injection for women with induced abortion. However, our study also has a few significant limitations. First, the trials included suffered from a high risk of bias, and only two trials clearly stated the method of random sequence generation. Second, we were unable to conduct a subgroup analysis to explore the source of heterogeneity for a limited number of studies. Most of the trials included in our analyses had small sample sizes and resulted in an imprecise estimation of effects with very low quality. Third, the trials we included were all conducted in China mainland and included women who were in the first termination; and no trials on the use of motherwort injection in women with intermediate or late abortions. Fourth, the most appropriate dosing schedule is still unknown for the limited evidence we found.

## 5 Conclusion

In conclusion, due to the small number of events and sample sizes and severe limitations, the current body of evidence is inadequate to establish the positive effects—including blood loss, duration of blood time, adverse events, the recovery time of normal menstruation and endometrial thickness—of motherwort injection preventing hemorrhage after abortion. Given the insufficiently high quality of these trials, future adequately powered, well-designed, and conducted trials are warranted to test the effects of the different treatments preventing hemorrhage fairly. Observational studies that carefully collect and analyze the data may also provide important insights regarding the effects of motherwort injection.

## Data Availability

The original contributions presented in the study are included in the article/Supplementary Material, further inquiries can be directed to the corresponding author.
