# Supplementary material for: Motherwort Injection for Preventing Uterine Hemorrhage in Women With Induced Abortion: A Systematic Review and Meta-Analysis of Randomized Evidence
Source: Front Pharmacol. 2022 Jul 21;13:916665. doi: 10.3389/fphar.2022.916665 (PMC9349354; doi:10.3389/fphar.2022.916665)
Supplement: Supplementary file 2 [file Table2.DOCX]

**Appendix1 Search Strategy**

**PubMed:**

#1 “Abortion, Induced” [MeSH Terms] 41058

#2 “induced abortion” [Title] 1782

#3 “Abortions, Induced” [Text Word] 103

#4 “Induced Abortions” [Title] 301

#5 abortion [Title] 24639

#6 abortions [Title] 3491

#7 “Abortion Technique*” [Text Word] 91

#8 “Technique*, Abortion” [Title] 965

#9 “Abortion Technic*” [Text Word] 16

#10 “Technic*, Abortion” [Title/Abstract] 838

#11 “Abortion*, Drug-Induced” [Title/Abstract] 806

#12 “Drug-Induced Abortion*” [Text Word] 34

#13 “therapeutic abortion” [Title] 749

#14 “uterine evacuation*” [Text Word] 425

#15 “medical abortion” [Text Word] 1526

#16 “surgical abortion” [Text Word] 680

#17 “first trimester abortion” [Text Word] 618

#18 #1 OR #2 OR #3 OR #4 OR #5 OR #6 OR #7 OR #8 OR #9 OR #10 OR #11 OR. #12 OR #13 OR #14 OR #15 OR #16 OR #17 52318

#19 Leonurus[MeSH Terms] 210

#20 leonuri*[Text Word] 198

#21 motherwort*[all field] 94

#22 #19 OR #20 OR #21 400

#23 #18 AND #22 6

**EMbase:**

#1 exp induced abortion/ 29792

#2 abortion induction.mp. 97

#3 abortion, induced.ti,ab. 198

#4 abortus provocatus.mp. 49

#5 artificial abortion.mp. 410

#6 provoked abortion.mp. 22

#7 pregnancy interruption.mp. 324

#8 #1 OR #2 OR #3 OR #4 OR #5 OR #6 OR #7

#9 exp Leonurus japonicus/ 93

#10 Chinese motherwort.mp. 17

#11 Leonurus artemisia.mp. 19

#12 Leonurus heterophyllus.mp. 65

#13 yi mu cao.mp. 17

#14 yimu cao.mp. 4

#15 yimucao.mp. 30

#16 #9 OR #10 OR #11 OR #12 OR #13 OR #14 OR #15 222

#17 #8 AND #16 2

**CENTRAL:**

#1 MeSH descriptor: [Leonurus] 3

#2 motherwort 18

#3 motherworts 0

#4 #1 OR #2 OR #3

#5 MeSH descriptor: [Abortion, Induced] 1136

#6 Induced Abortion* 1684

#7 Abortions, Induced 332

#8 Abortion* (Induced) 1684

#9 Technique, Abortion 325

#10 Abortions, Drug-Induced 14

#11 Abortion, Drug-Induced 57

#12 Drug-Induced Abortion* 61

#13 Abortion, Drug Induced 878

#14 Abortion Technique 325

#15 Techniques, Abortion 338

#16 Technics, Abortion 0

#17 Abortion Technics 0

#18 Technic, Abortion 5

#19 Abortion Techniques 338

#20 Abortion Technic 5

#21 #5 OR #6 OR #7 OR #8 OR #9 OR #10 OR #11 OR #12 OR #13 OR #14 OR #15 OR #16 OR #17 OR 18 OR #19 OR #20 2075

#22 #4 AND #21 1

CNKI ：(人工流产 + 流产 + 药物流产 + 清宫术 + 人流手术 + 人流 + 终止妊娠)*益母草 713

WanFang：（人工流产 OR 流产 OR 药物流产 OR 人流手术 OR 清宫术 OR 终止妊娠）AND 益母草 1095
